# Knowledge and Attitude of Nigerian Pregnant Women towards Antenatal Exercise: A Cross-Sectional Survey

**DOI:** 10.1155/2014/260539

**Published:** 2014-04-14

**Authors:** Chidozie E. Mbada, Olubukayomi E. Adebayo, Adebanjo B. Adeyemi, Olujide O. Arije, Olumide O. Dada, Olabisi A. Akinwande, Taofeek O. Awotidebe, Ibidun A. Alonge

**Affiliations:** ^1^Department of Medical Rehabilitation, College of Health Sciences, Obafemi Awolowo University, Ile-Ife, Nigeria; ^2^Departments of Obstetrics, Gynaecology & Perinatology, College of Health Sciences, Obafemi Awolowo University, Ile-Ife, Nigeria; ^3^Departments of Community Health, Obafemi Awolowo University Teaching Hospitals Complex, Ile-Ife, Nigeria; ^4^Department of Physiotherapy, University Health Services, University of Ibadan, Ibadan, Nigeria; ^5^Department of Physiotherapy, University College Hospital, Ibadan, Nigeria

## Abstract

*Background.* Engagement in physical exercise in pregnancy is hamstrung by safety concerns, skepticism about usefulness, and limited individualized prescription guidelines. This study assessed knowledge and attitude of pregnant women towards antenatal exercises (ANEx). *Methods*. The cross-sectional study recruited 189 pregnant women from six selected antenatal clinics in Ile-Ife, South-West, Nigeria. Data were obtained on maternal characteristics, knowledge, and attitude towards ANEx. *Results*. Relaxation and breathing (59.8%), back care (51.3%), and muscle strengthening (51.3%) exercises were the most commonly known ANEx. Prevention of back pain risk (75.9%) and excess weight gain (69.1%) were perceived as benefits, while lower extremities swelling (31.8%) and extreme weight gain or loss (30.7%) were considered as contraindications to ANEx. 15.8% of the respondents had negative attitude towards ANEx resulting from insufficient information on exercise (83.3%) and tiredness (70.0%). Age significantly influences knowledge about contraindications to ANEx (*P* = 0.001), while attitude was influenced by age and occupation, respectively (*P* < 0.05). There was significant association between attitude and knowledge about benefits and contraindications to ANEx (*P* < 0.05). *Conclusion.* A majority of Nigerian pregnant women demonstrated inadequate knowledge but had positive attitude towards ANEx. Knowledge about benefits and contraindications to ANEx significantly influenced the attitude towards exercise in pregnancy.

## 1. Background

Safe maternity with improved neonatal outcomes is predicated on proper antenatal care services [1, 2]. Exercise has become a fundamental aspect of women's lives and an important constituent of antenatal care [3–5]. Wang and Apgar [6] submitted that empirical data on the impact of exercise on the mother, the fetus, and the course of pregnancy are still limited and results of the few studies in humans are often equivocal or contradictory. However, the American Congress of Obstetricians and Gynaecologists [3] recommended that pregnant women can exercise moderately for 30 minutes on most days of the week. In accordance with these recommendations, irrespective of the pregnant woman's physical fitness level, exercise should be low-impact, moderate-intensity, and regular [3, 7].

Studies have recommended that women should initiate or continue exercise in most pregnancies [3, 4, 7] as it is safe for mother and not harmful to the foetus [3, 8, 9]. The health benefits of regular physical exercise in pregnancy include maintenance and improvement of physical fitness and cardiovascular endurance [4], prevention of excessive gestational weight gain and glucose intolerance [10, 11], conditioning of the muscles needed to facilitate labour [7, 12, 13], and improvement in psychological adjustment to changes in pregnancy [4]. Furthermore, exercise in pregnancy is correlated with a decrease in many common problems of pregnancy [14] and the stress of exercises produces certain adaptation such as healthier placenta and increased ability to deal with short decrease in oxygen [15].

In spite of the fact that exercise programs during pregnancy and after childbirth are designed to minimize impairment and help the woman maintain or regain function while she is preparing for the arrival of the baby and then caring for the infant [3, 16, 17], it is submitted that women are not meeting the exercise recommendations of the previous studies [3, 18, 19]. A myriad of factors not limited to beliefs and attitudes of women with respect to exercise in pregnancy [7, 20–22], level of knowledge [19, 22, 23], level of education [7], safety concern of the pregnant woman and her physician [6], race/ethnicity, and previous involvement in regular exercise have been implicated as important factors predisposing to exercise engagement or phobia among pregnant women. Thornton et al. [24] submitted that identifying factors that affect beliefs and behaviors would objectively encourage a change in attitude. Therefore, an assessment of knowledge and attitude about exercise in pregnancy may help to determine whether or not women will participate in exercise during and after pregnancy. This study was designed to assess knowledge and attitude of Nigerian pregnant women towards antenatal exercises.

## 2. Methods

One hundred and eighty-nine pregnant women were consecutively recruited into this cross-sectional survey. The respondents were recruited from six selected hospitals, namely, Urban Comprehensive Health Centre, Enuwa Primary Health centre, Comprehensive Health centre Aderemi, Obafemi Awolowo University (OAU) Health Centre, OAU Teaching Hospitals Complex, and Seventh Day Adventist Hospital in Ile-Ife, Osun state, South-West, Nigeria. Ethical approval for the study was obtained from the Health Research Ethics Committee of the Institute of Public Health, OAU, Ile-Ife, Nigeria (IPHOAU/12/13). Informed consent of all respondents was required for participation in the study.

The instrument for the study was an adapted questionnaire from a previous study by Ribeiro and Milanez [7] and was validated by expert reviews in a pilot study. The self-administered questionnaire sought information on sociodemographic, knowledge and attitude towards exercise in pregnancy. The Yoruba version (the local language spoken in the area where the study was conducted) of the questionnaire was administered to respondents who were not literate in English. The reliability of the Yoruba version of the questionnaire was assessed by a test-retest method (by observing 7 days between test and retest) among 10 pregnant women attending the UCHC of the OAUTHC, Eleyele, Ile-Ife. The summation of all the checked items on the questionnaire at test and retest was compared. The questionnaire items yielded an agreement percentage that ranged from 87.4 to 99.6%, the intraclass coefficient was 0.985, and the confidence interval ranged from 0.94 to 0.996. Pregnant women who were not literate in either English or Yoruba were excluded from the study.

### 2.1. Data Analysis

Descriptive statistics of mean, standard deviation, and frequency distribution were used to summarize data. Inferential statistics of the Chi-square test was used to test the associations between knowledge and attitude of women towards antenatal exercises and the respondents' characteristics. Alpha level was set at 0.05. Data was analyzed using Statistical Package for Social Sciences software version 16.0 (SPSS Inc., Chicago, USA).

## 3. Results

One hundred and eighty-nine respondents participated in this study. The mean age of the respondents was 28.9 ± 4.63 years. The sociodemographic characteristics of respondents are presented in Table 1. The result shows that the respondents were preponderantly of Christian religion (76.7%) and were traders or business women (54.5%). A majority of the respondents had tertiary education (69.4%) and were within the level of income of $100 to $200 per month (27.0%). The maternal-obstetrics characteristics of respondents are presented in Table 2. A majority of the respondents were nulliparous (39.2%) and commenced antenatal care within 1 to 3 months of pregnancy (28.6%).

Respondents had knowledge of pelvic floor exercise (37.0%), muscle strengthening exercise (51.3%), back care exercise (51.3%), and relaxation and breathing exercise (59.8%), respectively, as types of antenatal exercise (Table 3). However, swimming (21.7%) and cycling (20.6%) were the least known types of exercises in pregnancy.  Table 4 shows the knowledge of respondents' on benefits of and contraindications to antenatal exercises. Most of the respondents agreed that exercise in pregnancy would lead to reduction in risk of back pain (75.9%), prevention of excess weight gain (69.1%), and increased ability to cope with labour and delivery (69.6). On the other hand, lower extremities swelling (31.8%), extreme weight gain or loss (30.7%), and back pain (28.5%) during pregnancy were mostly considered as contraindications to exercise during pregnancy. The summative knowledge score revealed that 47.6% of the respondents had below average knowledge and 5.82% had average knowledge, while 46.6% had good knowledge of antenatal exercises.

Fifteen point eight percent of the respondents had negative attitude towards exercise. Lack of feeling to exercise (63.3%), tiredness (70.0%), and insufficient information on exercise (83.3%) were the most implicated factors for negative attitude towards antenatal exercises (Table 5). There was no significant association between knowledge about benefits of antenatal exercises and respondents characteristics (*P* > 0.05) (Table 6). However, there was significant association between knowledge about contraindications to antenatal exercises and age (*P* = 0.001) (Table 7).  Table 8 shows that age (*P* = 0.035) and occupation (*P* = 0.034) significantly influence attitude towards exercise in pregnancy. Furthermore, Chi-square test of association revealed a significant association between attitude and each piece of knowledge about benefit of (*P* = 0.001) and contraindication to (*P* = 0.001) antenatal exercise (Table 9).

## 4. Discussion

This study assessed knowledge and attitude of Nigerian pregnant women towards antenatal exercises. The women in this study were generally young and were mostly Christians and traders or business persons. A majority of the women had tertiary education and were within the level of income of $100 to $200 per month (27.0%). It has been found from previous studies that subjects' characteristics such as age [25], level of education [7, 19], and experience in infant and maternal issues [26] significantly influence knowledge, attitude, and perceptions of mothers towards exercises. Furthermore, most of the women in this study were nulliparous and commenced antenatal care within 1 to 3 months of pregnancy. This study's result on antenatal care commencement time is at variance with earlier findings of late antenatal care commencement among Nigerian [27–30] and other pregnant women from sub-Saharan Africa countries [31, 32]. The study's finding on antenatal care commencement time reflects an improvement in care seeking in pregnancy among Nigerian women. Therefore, it is believed that the findings of this study may have been influenced by the maternal sociodemographic characteristics.

Most of the women in this study had knowledge of pelvic floor exercise, muscle strengthening exercise, back care exercise, and relaxation and breathing exercise as types of antenatal exercises. However, swimming and cycling were mostly not known as types of antenatal exercises. Conversely, the American Pregnancy Association [33] ranked exercises in pregnancy in order as kegel, swimming, walking, bicycling, aerobics, and dance. It is adducible that the low level of knowledge of swimming among women in this study may not be unconnected with prevalent hydrophobia and cultural myths that makes swimming among pregnant women a taboo. In addition, lack of swimming skills and limited availability of swimming pools may have contributed to low level of knowledge of swimming as an important antenatal exercise. Furthermore, cycling or riding a stationary bike is a far-flung antenatal exercise in the study setting and could be linked to nonavailability or nonaffordability of bicycle ergometer for personal use. More so, it is not advisable for pregnant women to ride conventional bicycles on most Nigerian roads as there are no dedicated bikeways.

With regard to knowledge about effect of exercise on pregnancy, most of the women in this study believe that antenatal exercise reduces risk of back pain, promotes better ability to cope with labour and delivery, and prevents excessive weight gain. These findings are consistent with previous reports [12, 34, 35]. In contrast to the American College of Obstetrician and Gynecologists [3] recommendation on contraindication to antenatal exercise, the women in this study mostly implicated swelling of the lower extremities, extreme weight gain or loss, and presence of back pain during pregnancy as contraindications to exercise during pregnancy. These conditions are at best relative contraindications which should not rule out engagement in exercise during pregnancy except there are underlying medical or obstetric complications. However, there is paucity of data on contraindications to exercise during pregnancy. Although, there appears to be lack of evidence on why pregnant women without medical conditions should not be allowed to engage in exercise, but some level of caution is needed in the presence of some respiratory conditions [36, 37] or orthopedic conditions such as back and hip pain or joint problems [12, 38, 39]. Nonetheless, the result of this study revealed that the knowledge about benefits of antenatal exercises was not influenced by maternal sociodemographic characteristics. However, age was found to significantly influence knowledge about contraindications to antenatal exercises.

About 16% of the women in this study demonstrated negative attitude towards exercise in pregnancy. Therefore, a majority of the study samples seem to have positive attitude towards antenatal exercises in pregnancy. This finding is in tandem with recent studies that have reported a positive paradigm shift in attitudes toward exercise during pregnancy over the past two decades with increasing numbers of pregnant women participating in physical activities, exercises, and sports activities [3–5]. Improved knowledge of safety of exercise for both the mother and fetus during pregnancy in most cases has been linked to the willingness to initiate or continue antenatal exercises [3–5]. It was found in this study that attitude towards exercise in pregnancy was influenced mostly by tiredness, lack of feeling to exercise, and insufficient information on exercise. Similar findings have been reported by other authors [7, 19, 20, 40]. Specifically, in the study by Duncombe et al. [21] the most reported reasons for not exercising during pregnancy included feeling too tired, uncomfortable or sick and being busy. Ribeiro and Milanez [7] submitted that the fact that the principal barriers to exercising described by the pregnant women were lack of time and feeling tired and uncomfortable may suggest that many women do not feel motivated to exercise despite being aware of the possible benefits that physical exercise could offer to their health and the health of their baby. However, the result of this study revealed that the age and occupation significantly influence attitude towards antenatal exercises in pregnancy. Furthermore, knowledge about benefit of and contraindication to antenatal exercise significantly influenced the attitude of the women towards exercise in pregnancy. This finding is consistent with previous reports that revealed significant association between adequate knowledge of antenatal exercises and attitudes toward exercise during pregnancy [7, 22, 41].

This study provides an empirical data on knowledge and attitude of Nigerian pregnant women towards exercise in pregnancy. Hitherto, there is an apparent dearth of studies on exercise culture of women in sub-Sahara Africa. The outcomes of this study underscore the need of health education programmes on the importance of exercise in pregnancy among women from sub-Sahara Africa countries. Physical exercise plays a significant role in maternal health and creating awareness of its benefit and contraindications among local women of childbearing age will improve engagement in and attitude towards exercise, improve maternal outcomes, and eventually decrease the burden of pregnancy-related preventable conditions on the health care system. However, the outcome of this study is limited in its generalizability and needs to be validated in other settings.

## 5. Conclusion

A majority of Nigerian pregnant women demonstrated inadequate knowledge about antenatal exercises. However, the women had positive attitude towards exercise. Knowledge about benefit of and contraindication to antenatal exercise significantly influenced the attitude towards exercise in pregnancy.

## Figures and Tables

**Table 1:** Sociodemographic characteristics of the respondents (*N* = 189).

Variable	*n* (%)
Religion	
Christianity	145 (76.7)
Islam	43 (22.8)
Traditional religion	1 (0.5)
Occupation	
Home maker	23 (12.2)
Trading/business	103 (54.5)
Civil/public service	39 (20.6)
Schooling	20 (10.6)
Not specified	4 (2.1)
Educational qualification	
Primary	9 (4.8)
Secondary	49 (25.9)
Tertiary	131 (69.4)
Income	
Less than $100	32 (16.9)
$100–$200	51 (27.0)
$200–$300	38 (20.1)
$300–$500	13 (6.9)
$500–$1000	10 (5.3)
Greater than $1000	10 (5.3)
No Disclosure	25 (13.2)

**Table 2:** Maternal-obstetric characteristics of the respondents (*N* = 189).

Variable	*n* (%)
Family setting	
Polygamy	38 (20.1)
Monogamy	125 (66.1)
Single parenting	26 (13.8)
Parity	
Nulliparous	74 (39.2%)
Primiparous	53 (28.0%)
Multiparous	62 (32.8%)
Mode of delivery	
Vaginal	109 (57.6%)
Caesarean section	6 (3.2%)
Not applicable	74 (39.2%)
Place of delivery	
Hospital	93 (49.2%)
Home	7 (3.7%)
Mission	15 (7.9%)
Not applicable	74 (39.2%)
Previous antenatal care start time	
<1 month	5 (2.6%)
1–3 months	54 (28.6%)
3–6 months	44 (23.3%)
6–9 months	12 (6.3%)
Not applicable	74 (39.2%)
Present antenatal care duration	
<1 month	75 (39.7%)
1–3 months	57 (30.2%)
3–6 months	44 (23.2%)
6–9 months	13 (6.9%)

**Table 3:** Knowledge of respondents on different types of exercises in pregnancy (*N* = 189).

Variable	Yes	No
	*n* (%)	*n* (%)
Aerobics	59 (31.2)	130 (68.8)
Pelvic floor exercise	70 (37.0)	119 (63.0)
Swimming	41 (21.7)	148 (78.3)
Stretching exercise	76 (40.2)	113 (59.8)
Muscle strengthening exercise	97 (51.3)	92 (48.7)
Abdominal exercise	56 (29.6)	133 (70.4)
Back care exercise	97 (51.3)	92 (48.7)
Cycling	39 (20.6)	150 (79.4)
Relaxation and breathing exercise	113 (59.8)	76 (40.2)

**Table 4:** Knowledge of respondents' on benefits of and contraindications to antenatal exercises (*N* = 189).

Items	Agree (%)	I (%)	DG (%)	AS (%)
*Benefits *				
(1) Reduces risk of back pain during pregnancy	75.9	18.4	5.8	84
(2) Prevents excessive weight gain during pregnancy	69.1	23.3	7.7	79
(3) Increases risk of urinary incontinence during pregnancy	41.3	43.8	14.7	69
(4) Increases risk of diabetes during pregnancy	35.1	47.7	17.2	65
(5) Strengthens pelvic floor muscles during pregnancy	54.3	37.0	8.8	73
(6) Increases formation of varicose veins during pregnancy	35.3	55.9	8.8	68
(7) Increases risk of swelling of extremities during pregnancy	56.9	37.3	5.8	76
(8) Causes high blood pressure during pregnancy	58.4	35.1	6.6	76
(9) Increases muscle tone, strength, and endurance during pregnancy	59.5	34.0	6.6	78
(10) Increased energy and stamina during pregnancy	64.1	30.1	5.7	79
(11) Improvement of body awareness, posture, coordination, and balance during pregnancy	63.8	31.5	4.6	80
(12) Better ability to cope with labour and delivery	69.6	27.4	3.0	82
(13) More rapid postnatal recovery	67.4	30.1	2.5	80
*Contraindications *				
(1) Vaginal bleeding during pregnancy	14.3	34.2	51.5	73
(2) Uterine contractions during pregnancy	17.8	38.1	44.1	70
(3) Chest pain during pregnancy	15.3	40.3	44.3	70
(4) Migraine during pregnancy	12.1	43.0	44.9	71
(5) Difficulty in breathing during pregnancy	16.5	38.9	44.6	70
(6) Swelling of the extremities during pregnancy	31.8	42.2	26	62
(7) Back pain during pregnancy	28.5	45.8	25.7	61
(8) Extreme weight gain or loss during pregnancy	30.7	48.2	21.1	57
(9) Abdominal pain during pregnancy	20.8	40.8	38.4	65
(10) Muscle weakness during pregnancy	33.9	38.9	27.1	62
(11) Dizziness during pregnancy	17.3	42.5	40.3	68
(12) Diabetes during pregnancy	15.6	44.7	39.7	54
(13) Premature labour during pregnancy	10.9	41.9	47.1	73

Key: I: indifferent; DG: disagree; AS: average Score.

**Table 5:** Attitude of respondents towards antenatal exercises.

Variable	*n* (%)
Attitude towards antenatal exercises (*N* = 189)	
Positive	159 (84.1)
Negative	30 (15.8)
Factors influencing negative attitude antenatal exercises (*n* = 30)	
I feel tired to exercise	21 (70.0)
I do not feel like exercising	19 (63.3)
I have busy schedule	9 (30.0)
I have a lot of child care activities	6 (20.0)
I am afraid of exercise	5 (16.6)
I do not have sufficient information on exercise	25 (83.3)

**Table 6:** Chi-square test of association between knowledge about benefits of antenatal exercises and respondents characteristics.

	BAK (*n* = 90)	AK (*n* = 11)	AAK (*n* = 88)	*X* ^2^	*P*-value
Age group					
<30 years	60 (66.7%)	3 (27.3%)	55 (58.3%)	7.47	0.058
≥30 years	30 (33.3%)	8 (72.7%)	33 (41.7%)		
Educational qualification					
Primary	10 (11.1%)	3 (27.3%)	5 (5.7%)	8.53	0.202
Secondary	26 (28.9%)	2 (18.2%)	23 (26.1%)		
Tertiary	54 (60%)	6 (54.5%)	60 (68.2%)		
Occupation					
Home maker	11 (12.6%)	2 (18.2%)	11 (12.5%)	6.98	0.859
Trading/business	45 (50.0%)	4 (36.4%)	44 (50.0%)		
Civil/public service	17 (18.9%)	4 (36.4%)	22 (25.0%)		
Schooling	13 (14.4%)	1 (9.1%)	7 (8.0%)		
Not specified	4 (4.4%)	0 (0%)	4 (4.5%)		
Parity					
Nulliparous	19 (21.1%)	1 (9.1%)	19 (21.6%)	8.15	0.227
Primiparous	35 (38.9%)	1 (9.1%)	31 (35.2%)		
Multiparous	36 (40.0%)	9 (81.8%)	38 (43.2%)		

Key: BAK: below average knowledge; AK: average knowledge; AAK: above average knowledge.

Significance at **α** = 0.05.

**Table 7:** Chi-square test of association between knowledge about contraindications to antenatal exercises and respondents characteristics.

	BAK (*n* = 90)	AK (*n* = 11)	AAK (*n* = 88)	*X* ^2^	*P*-value
Age group					
<30 years	65 (62.5%)	5 (45.5%)	52 (61%)	17.03	0.001
≥30 years	39 (37.5%)	6 (54.5%)	36 (39%)		
Educational qualification					
Primary	2 (2.2%)	2 (18.2%)	6 (6.8%)	9.58	0.143
Secondary	27 (30.0%)	3 (27.3%)	20 (22.7%)		
Tertiary	61 (67.8%)	6 (54.5%)	62 (70.5%)		
Occupation					
Home maker	15 (16.7%)	1 (9.1%)	14 (15.9%)	7.43	0.828
Trading/business	36 (40.0%)	8 (72.7%)	40 (45.5%)		
Civil/public service	25 (27.8%)	1 (9.1%)	20 (22.7%)		
Schooling	11 (12.2%)	1 (9.1%)	9 (10.2%)		
Not specified	3 (3.3%)	0 (0%)	5 (5.7%)		
Parity					
Nulliparous	21 (21.5%)	3 (27.3%)	14 (15.9%)	10.45	0.107
Primiparous	38 (38.5%)	1 (9.1%)	33 (37.5%)		
Multiparous	31 (40%)	7 (63.6%)	41 (46.6%)		

Key: BAK: below average knowledge; AK: average knowledge; AAK: above average knowledge.

**Table 8:** Chi-square test of association between attitude of respondents towards exercise and respondents' characteristics.

	NA (*n* = 30)	PA (*n* = 159)	*X* ^2^	*P*-value
Age group				
<30 years	24 (74.5%)	95 (59.4%)	4.439	0.035*
≥30 years	6 (25.5%)	64 (40.6%)		
Educational qualification				
Primary	3 (10.0%)	7 (4.4%)	5.708	0.058
Secondary	12 (40.0%)	38 (23.9%)		
Tertiary	15 (50.0%)	114 (71.7%)		
Occupation				
Home maker	8 (26.7%)	17 (10.7%)	10.400	0.034*
Trading/business	12 (40.0%)	82 (51.6%)		
Civil/public service	3 (10.0%)	38 (23.9%)		
Schooling	6 (20.0%)	15 (9.4%)		
Not specified	1 (3.3%)	7 (4.4%)		
Parity				
Nulliparous	5 (16.7%)	33 (20.8%)	2.582	0.275
Primiparous	15 (50.0%)	55 (34.6%)		
Multiparous	10 (33.3%)	71 (44.7%)		

Key: NA: negative attitude; PA: positive attitude; *Significance at **α** = 0.05.

**Table 9:** Chi-square test of association between attitude and knowledge about antenatal exercise.

Value	NA (*n* = 30)	PA (*n* = 159)	*X* ^2^	*P*-value
Benefits of antenatal exercises				
Below average knowledge	24 (80.0%)	67 (42.1%)	16.645	0.001*
Average knowledge	2 (6.7%)	11 (6.9%)		
Above average knowledge	4 (13.3%)	86 (54.1%)		
Contraindications to antenatal exercises				
Below average knowledge	23 (76.7%)	68 (42.8%)	13.305	0.001*
Average knowledge	2 (6.7%)	7 (4.4%)		
Above average knowledge	5 (16.7%)	84 (52.8%)		

Key: NA: negative attitude; PA: positive attitude. *Significance at **α** = 0.05.
